# Photocatalytic Degradation of Palm Oil Mill Effluent (POME) Waste Using BiVO_4_ Based Catalysts

**DOI:** 10.3390/molecules26206225

**Published:** 2021-10-15

**Authors:** Wibawa Hendra Saputera, Aryan Fathoni Amri, Rino R. Mukti, Veinardi Suendo, Hary Devianto, Dwiwahju Sasongko

**Affiliations:** 1 Research Group on Energy and Chemical Engineering Processing System, Department of Chemical Engineering, Faculty of Industrial Technology, Institut Teknologi Bandung, Jl. Ganesha No. 10, Bandung 40132, Indonesia; aryanfathoni@gmail.com (A.F.A.); hardev@che.itb.ac.id (H.D.); sasongko@che.itb.ac.id (D.S.); 2 Center for Catalysis and Reaction Engineering, Institut Teknologi Bandung, Jl. Ganesha No. 10, Bandung 40132, Indonesia; rino@chem.itb.ac.id; 3 Research Center for New and Renewable Energy, Institut Teknologi Bandung, Jl. Ganesha No. 10, Bandung 40132, Indonesia; 4 Division of Inorganic and Physical Chemistry, Faculty of Mathematics and Natural Sciences, Institut Teknologi Bandung, Jl. Ganesha No. 10, Bandung 40132, Indonesia; vsuendo@chem.itb.ac.id; 5 Research Center for Nanoscience and Nanotechnology, Institut Teknologi Bandung, Jl. Ganesha No. 10, Bandung 40132, Indonesia

**Keywords:** palm oil mill effluent (POME), photocatalytic degradation, BiVO_4_, defects, vanadium vacancy

## Abstract

Disposal of palm oil mill effluent (POME), which is highly polluting from the palm oil industry, needs to be handled properly to minimize the harmful impact on the surrounding environment. Photocatalytic technology is one of the advanced technologies that can be developed due to its low operating costs, as well as being sustainable, renewable, and environmentally friendly. This paper reports on the photocatalytic degradation of palm oil mill effluent (POME) using a BiVO_4_ photocatalyst under UV-visible light irradiation. BiVO_4_ photocatalysts were synthesized via sol-gel method and their physical and chemical properties were characterized using several characterization tools including X-ray diffraction (XRD), scanning electron microscopy (SEM), transmission electron microscopy (TEM), surface area analysis using the BET method, Raman spectroscopy, electron paramagnetic resonance (EPR), and UV-Vis diffuse reflectance spectroscopy (UV-Vis DRS). The effect of calcination temperature on the properties and photocatalytic performance for POME degradation using BiVO_4_ photocatalyst was also studied. XRD characterization data show a phase transformation of BiVO_4_ from tetragonal to monoclinic phase at a temperature of 450 °C (BV-450). The defect site comprising of vanadium vacancy (V_v_) was generated through calcination under air and maxima at the BV-450 sample and proposed as the origin of the highest reaction rate constant (*k*) of photocatalytic POME removal among various calcination temperature treatments with a *k* value of 1.04 × 10^−3^ min^−1^. These findings provide design guidelines to develop efficient BiVO_4_-based photocatalyst through defect engineering for potential scalable photocatalytic organic pollutant degradation.

## 1. Introduction

The rapid growth of the palm oil industry worldwide has invited serious water pollution in aquatic systems. Large crude palm oil (CPO) production has resulted in an increase in palm oil mill effluent (POME) generated from the CPO process. Each ton of CPO production produces about 2.5–3.0 m^3^ of POME [1]. Huge amounts of POME with a high concentration of organic content have implications in environmental pollution if not handled properly. POME is declared as one of the most difficult wastes to handle due to its large production and ineffective processing technology.

Fresh POME is generally acidic (pH 3.3–4.6), high in temperature (60–80 °C), thick, brownish in color with solids (1330–50,700 mg/L), with oils and fats (190–14,720 mg/L), a biochemical oxygen demand (BOD) of 8200–35,000 mg/L, and chemical oxygen demand (COD) of 15,103–65,100 mg/L [2]. Regulations regarding quality standards for the discharge of POME into the environment or water bodies to prevent the negative effects of POME waste have been established. The latest regulations state that the COD and BOD standards were set at lower than 250 mg/L and 100 mg/L, respectively.

The most widely used POME waste treatment is conventional treatment using an open pond system through anaerobic and aerobic decomposition processes. The disadvantage of this system is that apart from requiring a large area of land and a long retention time, it can also emit harmful gases such as methane gas and cause sludge build-up [3]. Therefore, conventional treatment is very inefficient for treating POME. In recent years, various treatment methods have been developed to eliminate and reduce POME pollution [3]. These methods include composting [4], fermentation [5], coagulation flocculation [6], adsorption [7], flotation, membrane technology [8], steam reforming [9], and advanced oxidation processes [10]. Among these technologies, photocatalytic technology is a viable wastewater technology to reduce environmental pollution especially POME waste due to it being eco-friendly, not involving sludge formation and other harmful substances, as well as being cost effective, operated at ambient conditions, and a sustainable process, which addresses the issues of energy consumption and environmental remediation [11].

TiO_2_ is by far the most widely used photocatalyst due to it exhibiting strong oxidizing ability, stability, and high efficiency against organic compounds [12,13]. However, the main drawback of TiO_2_ is its wide band gap (~3.2 eV), which limits its light response to the UV region. As a result, this photocatalyst can only take advantage of less than 6% of the solar energy impinging on the Earth’s surface, and its potential as a sustainable technology cannot be entirely fulfilled [14]. Therefore, during the last few years an increasingly great number of new photocatalysts have been developed and tested as possible alternatives to TiO_2_ based photocatalysts. In this context, the feasibility of using some well-known photocatalysts like bismuth vanadate (BiVO_4_) has been reconsidered in light of recent advances in nanotechnology.

BiVO_4_ is a less expensive photocatalyst with a narrower band gap (2.4 eV) than TiO_2_, which is capable of harvesting visible light for photocatalytic processes [15,16]. BiVO_4_ has been reported to successfully degrade and reduce organic pollutants in wastewater, using methylene blue [17], rhodamine blue [18], and methyl orange [19] as model compounds. The photocatalytic efficiency is closely related to the structure, crystal dimensions, size, morphology, optical band-gap energy, and surface shape. It is also generally considered that the synthesis method and the operating conditions used were the main critical parameters to achieve optimum efficiency removal [17]. Several studies have been carried out on the synthesis of BiVO_4_ with various methods including coprecipitation [20], hydrothermal [21], and sol-gel methods [22]. Among these methods, the sol-gel method is becoming increasingly used because it requires only simple equipment and a low process temperature, compared to the traditional powder method [17]. However, up to now there has been no research that has developed a BiVO_4_ photocatalyst specifically for POME waste treatment.

Herein, we report the fabrication of BiVO_4_ through a simple sol-gel method. The as-prepared BiVO_4_ catalyst was employed for the first time for photocatalytic POME degradation. Furthermore, we have analyzed the as-prepared catalysts in detail by various spectroscopic and microscopic characterizations. The obtained photocatalytic rates for POME degradation will be discussed on the basis of characterization data which is highly dependent on the calcination temperature in the range of 300–600 °C. This is attributed to the variation in crystallinity, morphology, optical and electronic properties, and local structure distortion (existence of defect sites, namely vanadium vacancy) of the BiVO_4_ upon calcination treatment.

## 2. Results and Discussion

### 2.1. Crystal Phase Composition

**Figure 1:**
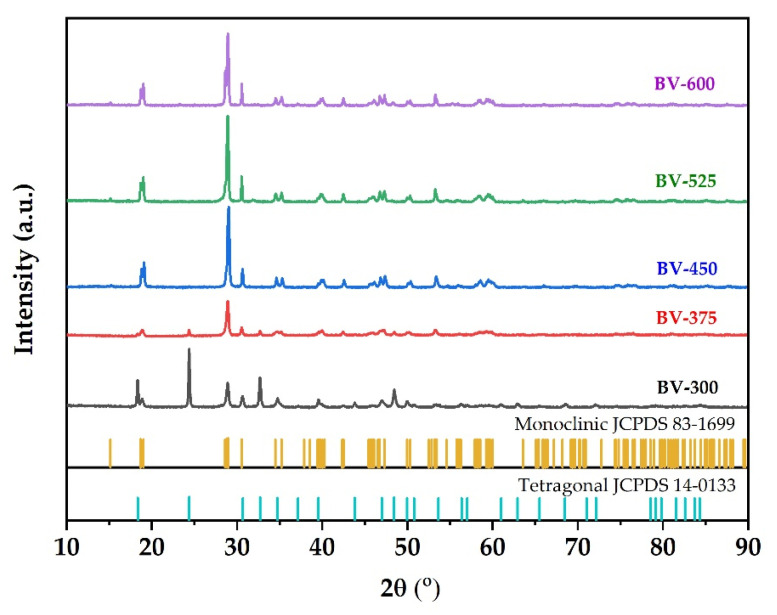
XRD pattern of BiVO_4_ photocatalyst with different calcination temperatures.

XRD is used to characterize the phase structure of the obtained samples. Figure 1 shows the XRD pattern of the synthesized BiVO_4_ photocatalyst at different calcination temperatures. Samples are labeled BV-x, where BV and x are attributed to BiVO_4_ and calcination temperature, respectively. The XRD pattern of BV-300 indicates that the sample is in a mixture of tetragonal and monoclinic phases. The diffraction peaks at 2θ = 18.3; 24.4; 32.7; 34.7; 39.5; 43.8, and 48.4 were attributed to the tetragonal structure of BiVO_4_ (JCPDS, no. 14-0133). Meanwhile, the diffraction peaks at 2θ = 18.7 and 28.9 were assigned to the monoclinic BiVO_4_ structure (JCPDS, no. 83-1699). When the calcination temperature was increased to 375 °C (BV-375), a phase change began to occur towards the monoclinic, which can be seen by decreasing the intensity of the tetragonal phase and followed by an increase in intensity in the monoclinic phase. When the calcination temperature increases to 450 °C (BV-450), the tetragonal phase peak disappeared while it predominantly consists of the monoclinic phase. In addition, it generated a sharper XRD pattern, which shows better crystallinity and an increase in crystallite size. Subsequent calcination to temperatures of 525 °C (BV-525) and 600 °C (BV-600) showed that the photocatalysts were all in a monoclinic phase (there were no peaks of tetragonal phase or other impurities were detected).

According to the Zhang et al. [17], when the calcination temperature was increased up to 400 °C, there were several small peaks at 2θ = 25.7°; 27.7°, and 32.3°, which correspond to Bi_2_O_3_ being considered an impurity. Increasing the calcination temperature to 450 °C causes the peak corresponding to Bi_2_O_3_ to disappear. The calcination temperature has a significant impact on the phase composition, degree of crystallinity, and photocatalytic properties of the prepared photocatalyst [23]. These results indicate that the calcination temperature is an important factor in the preparation of pure monoclinic BiVO_4_. The intensity of the XRD diffraction peak of BiVO_4_ photocatalyst after calcination at 525 °C was the strongest followed by 450 °C. The narrow and sharp diffraction peaks indicate that the BiVO_4_ photocatalyst has high crystallinity. This increase in photocatalyst crystallinity will be advantageous for photocatalytic activity.

On the other hand, the XRD results of the synthesized BiVO_4_ photocatalyst are different from Wang et al. [23], where the calcination was carried out at a temperature range of 350–500 °C for 5 h to produce a crystal structure of BiVO_4_ photocatalyst with a pure monoclinic phase structure. In addition, the intensity of the diffraction peak increased with increasing calcination time, indicating that the degree of crystallinity increased with the extension of the calcination time. However, if the calcination time is too long, the monoclinic phase transformed back to a tetragonal phase. In addition, Pookmanee et al. [24] reported that XRD pattern showed that all BiVO_4_ photocatalyst crystals with monoclinic phase structure at the calcination temperature treatment range of 400–600 °C for 2 h. No other phase peaks or impurities were detected. As the calcination temperature increases, the width of the diffraction line decreases and the intensity of the diffraction line increases indicating that a high crystallinity of BiVO_4_ photocatalyst was obtained.

The crystal size of the sample was calculated according to the Scherrer formula. The crystal sizes of the BV-300, BV-375, BV-450, BV-525, and BV-600 were 23.79; 21.58; 23.89; 25.68; 26.06 nm, respectively (Table 1). These results indicate that crystal size increases with increasing calcination temperature in general, which implies that high temperatures favor grain growth stages according to nucleation theory and thermodynamic growth [23]. However, the BV-375 photocatalyst was inversely proportional due to the phase transformation from the tetragonal phase to the monoclinic phase. The appearance of a broad peak in the BV-375 photocatalyst indicates a decrease in crystal size.

**Figure 2:**
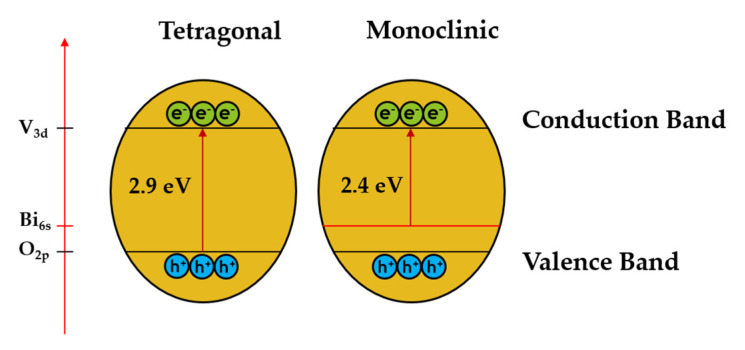
Schematic diagram of BiVO_4_ in two different crystal structures, namely tetragonal and monoclinic phases. Adapted from ref. [25].

It is known that the monoclinic BiVO_4_ crystal structure consists of O 2p and Bi 6s orbitals in the valence band and V 3d orbitals in the conduction band while the tetragonal structure of BiVO_4_ consists of only O 2p species in the valence band [25]. The presence of the Bi6s orbital in the valence band of BiVO_4_ is a regulatory factor that helps to improve charge separation and electron-hole pair migration, resulting in better photocatalytic performance (as shown in Figure 2).

**Figure 3:**
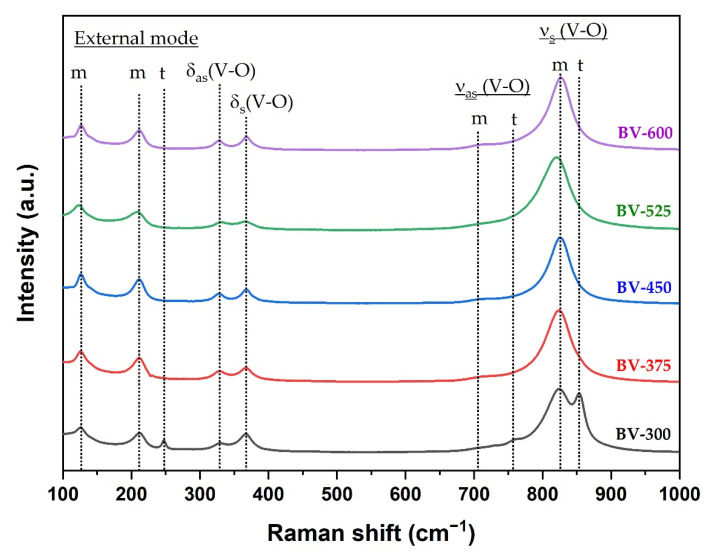
Raman spectra of the BiVO_4_ samples synthesized at different calcination temperatures where *m* and *t* are attributed to the monoclinic and tetragonal phases, respectively.

Raman spectroscopy can provide more structural information and is used to support the transformation phase from tetragonal to monoclinic phase by increasing the calcination temperature. The Raman spectrum of the BiVO_4_ photocatalyst with different calcination temperatures is shown in Figure 3. In the BiVO_4_ spectrum, the peaks at 827 cm^−1^ and 854 cm^−1^ correspond to the symmetrical V-O stretching mode of monoclinic and tetragonal phases, respectively. The weak shoulder peak near 710 cm^−1^ was designated as the monoclinic V-O asymmetric stretching mode, while the shoulder peak near 755 cm^−1^ was designated as the tetragonal phase. The double peaks observed at about 328 cm^−1^ and 366 cm^−1^ were associated with an asymmetric bending mode (VO_4_) and a symmetric bending mode (VO_4_) respectively. The peaks at 126 cm^−1^ and 210 cm^−1^ correspond to crystal lattice vibrations for the monoclinic phase while the peak at 247 cm^−1^ is attributed to the tetragonal phase (external mode). The Raman spectra clearly confirmed the phase transformation from tetragonal to monoclinic phase obtained from XRD measurement (Figure 1) at a calcination temperature of 375 °C (BV-375) indicated by the absence of the peaks at 247, 755, and 854 cm^−1^.

### 2.2. Morphology

**Figure 4:**
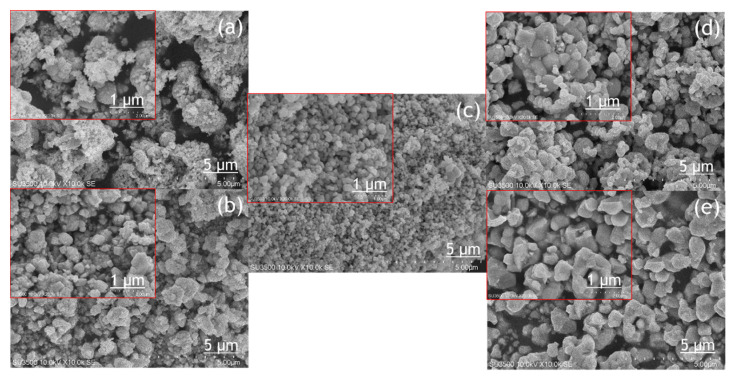
SEM image of the photocatalyst of BiVO_4_ photocatalysts: (**a**) BV-300, (**b**) BV-375, (**c**) BV-450, (**d**) BV-525, and (**e**) BV-600.

**Figure 5:**
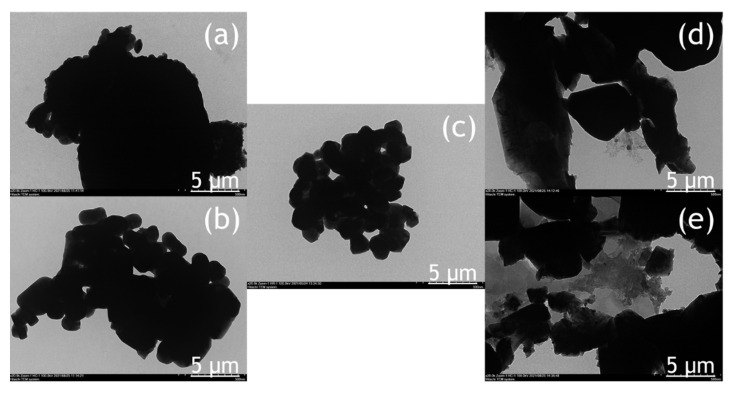
TEM image of the photocatalyst of BiVO_4_ nanoparticles: (**a**) BV-300, (**b**) BV-375, (**c**) BV-450, (**d**) BV-525, and (**e**) BV-600.

The morphology of the BiVO_4_ photocatalyst was investigated by SEM and TEM, as shown in Figure 4 and Figure 5. The tendency of agglomeration in the BiVO_4_ photocatalyst occurred more extensively at a low calcination temperature (BV-300) and high calcination temperature (BV-600) compared to the calcination temperature of 450 °C (BV-450). The average particle sizes for BV-300, BV-375, BV-450, BV-525, and BV-600 photocatalysts were 0.759, 0.429, 0.154, 0.425, and 0.709 µm, respectively. Based on Figure 4, with an increase in the calcination temperature up to 450 °C, the particle size decreased, while beyond that, the particle size gradually increased. The average particle size of the BV-600 photocatalyst from this study exhibited a similar size to the work reported by Pookmanee et al. [24]. Moreover, it was also revealed that uneven distribution of grains and shapes was observed in BV-300 and BV-375. When the calcination temperature rises up to 450 °C (BV-450), although the agglomeration phenomenon slightly occurs, the grain size is small and has a uniform distribution. The grains become larger at higher temperatures, providing sufficient energy leading to agglomeration. At a calcination temperature of 600 °C (BV-600), the resulting smaller grains gradually begin to melt and reform into larger particles. This phenomenon is in line with the “big eat small” phenomenon in the material sector. Morphological changes in this study are in accordance with the work reported by Wang et al. [18].

The TEM image (Figure 5) provides a clearer visualization of the morphology of the BiVO_4_ photocatalyst at different calcination temperatures. The BV-450 photocatalyst mainly consisted of small granules, while BV-300, BV-375, BV-525, and BV-600 exhibited irregular shapes. The estimated size of the BV-450 photocatalyst showed predominantly particles with sizes around 150 nm. The good morphology of BV-450 shows the feasibility of these nanostructures for photocatalytic applications.

The atomic compositions of the BiVO_4_ photocatalyst were further studied by energy-dispersive X-ray spectroscopy (EDX) and the corresponding results are shown in Table 2. For all photocatalysts obtained at different calcination temperatures, it consisted of Bi, V, and O with various composition percentages while no additional elements were detected, indicating that the chemical composition of the synthesized BiVO_4_ exhibited good product crystallinity. In addition, EDX analysis was carried out to ensure the consistency of the Bi/V ratio between the starting material and the final product within the error range of the experiment. The atomic ratio of the Bi/V trend fluctuated at different calcination temperatures, indicating that the size of the BiVO_4_ nanoparticles is not uniform and agglomeration occurs occasionally, which was also confirmed by TEM analysis (Figure 5). These results are also in line with those reported in previous studies [26]. In addition, the alteration of the Bi/V ratio indicated lattice distortion and thus generating defect sites on the bulk and/or surface of BiVO_4_ photocatalyst [27].

### 2.3. Adsorption–Desorption Isotherm Profile

**Figure 6:**
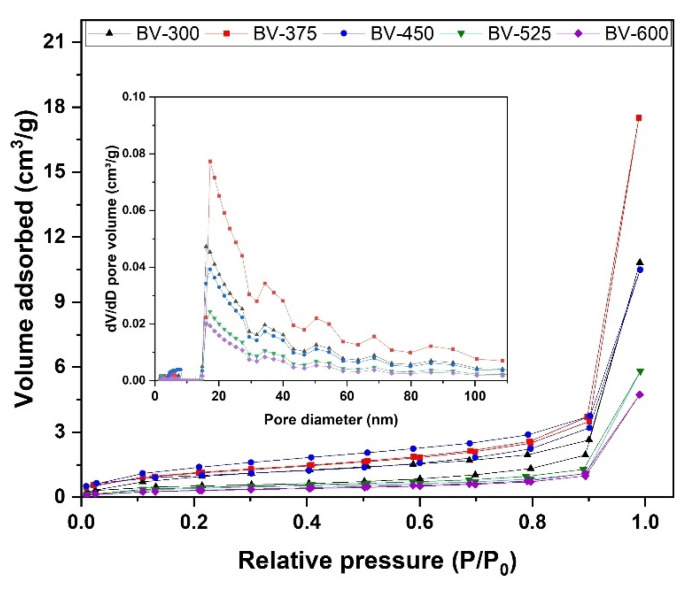
The N_2_ adsorption–desorption isotherm curve of BV-300, BV-375, BV-450, BV-525, and BV-600. Inset: the corresponding pore size distribution.

Brunauer–Emmett–Teller (BET) and Barrett–Joyner–Halenda (BJH) methods are effective methods for measuring the specific surface area and pore size of photocatalysts, respectively. Figure 6 shows the N_2_ adsorption and desorption isotherms of the BiVO_4_ photocatalyst and the corresponding pore size distribution curves (Figure 6, inset). The isotherms exhibit hysteresis loops with sharp adsorption and desorption branches at relatively high pressures. The physisorption isotherm can be categorized as type IV, representing mesoporous powders, which has been reported in previous studies as one of the clear advantages in degrading organic pollutants in water [28]. The pore size distribution can be calculated from the branch desorption isotherm using the BJH model. The results shown in Table 3 show that the BiVO_4_ photocatalysts exhibited the most likely pore sizes of around 19–22 nm. The calculated BET surface area for BiVO_4_ photocatalysts is in the range of 1.133–4.040 m^2^/g. In the ideal case when the particle size decreases, the surface area per unit volume (or mass) increases whereas the formation of porosity, especially in smaller pores, generates a much larger surface area than that produced by the reduction in particle size. The BV-450 photocatalyst exhibits a larger BET surface area than BV-300 and BV-600 due to its smaller particle size and pore size which will be beneficial to enhance photocatalytic performance. These findings were in agreement with Zhang et al. [17] which reported that as-prepared BiVO_4_ catalyst exhibited specific surface area between 3.0 and 5.0 m^2^/g but with smaller pore size, in the range of 30–50 nm.

### 2.4. Optical Properties

**Figure 7:**
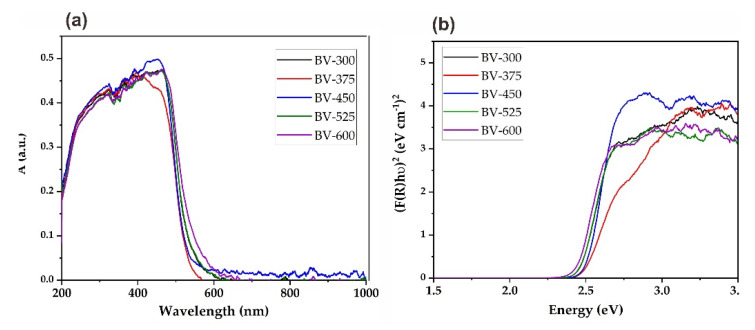
(**a**) UV-Vis DRS spectrum (**b**) band-gap energy estimation using the Kubelka–Munk F(R) function of BV-300, BV-375, BV-450, BV-525, and BV-600.

The optical absorption properties of the prepared BiVO_4_ photocatalyst with different calcination temperatures were investigated with UV-Vis diffuse reflectance spectroscopy (DRS) and the spectrum is presented in Figure 7. It showed that there was a slight shift towards the visible region by increasing the calcination temperature (Figure 7a). The corresponding graph to calculate the band-gap energy based on the Kubelka–Munk formula is presented in Figure 7b. The band-gap energy for the BiVO_4_ photocatalyst can be estimated between 2.44 and 2.50 eV (Table 4) indicating that the BiVO_4_ photocatalyst can work efficiently under visible light irradiation. These data also clearly show that the electronic structure of the BiVO_4_ photocatalyst changes due to the calcination temperature used in the sol-gel synthesis. Variations in the electronic structure lead to different degrees of delocalization of photogenerated electron-hole pairs, thereby resulting in different photogenerated electron-hole mobilites [29].

The increase in band-gap energy from photocatalyst BV-300 to BV-450 is due to a reduction of particle size, hence a shift in wavelength towards a shorter region (blue shift) was obtained. Meanwhile, the decrease in band-gap energy in the beyond calcination temperature of 450 °C is due to a larger particle size (agglomeration) and hence a shift in wavelength towards a longer region (red shift) was observed. These findings are in accordance with the previous work reported by Sun et al. [30]. The increase in calcination temperature will provide a new energy level that makes it easier for electrons to be excited from the valence band to the conduction band. However, there should be an optimum band-gap energy of BiVO_4_ that needs to be achieved since the smaller the band-gap energy, the easier the electron-hole pair recombination process will occur, so that the photocatalytic activity will be inhibited. Since BV-450 exhibited the widest band-gap energy among other catalysts, it might decrease the recombination rate of photogenerated electron-hole pairs. As a consequence, this would lead to an improvement in photoactivity. It should be noted that in addition to photo absorption, other factors are also significant and should be considered in achieving optimum photocatalytic efficiency of the photocatalyst, such as the electron-hole pair separation efficiency and the number of defect sites [16], especially for photocatalytic degradation of organic compounds.

### 2.5. Defect Sites

**Figure 8:**
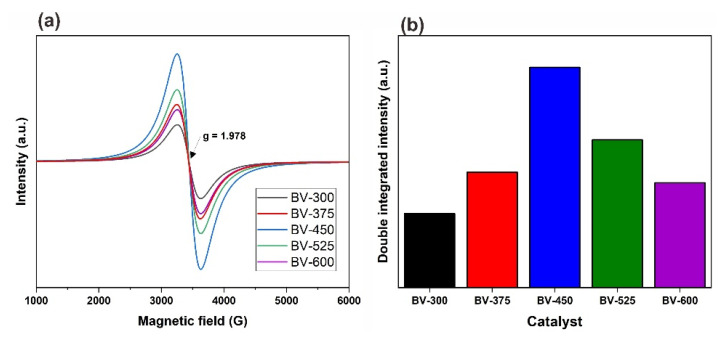
(**a**) EPR spectra acquired at 120 K of BV-300, BV-375, BV-450, BV-525, and BV-600. (**b**) Bar graphs representing the double integrated intensity of its corresponding EPR spectra.

Another critical property of BiVO_4_ that needs to be evaluated is the existence of defect sites in the form of V^4+^ associated with oxygen vacancies. Defect sites in BiVO_4_ can be produced from the evolution of oxygen and the volatilization of bismuth and vanadium oxides [31]. In addition, Lamers et al. reported that by purging as-synthesized BiVO_4_ photocatalyst with air and inert gas, such as argon and nitrogen, it could generate different types of defect sites, such as vanadium and oxygen vacancies [32]. Consequently, vanadium vacancies (V_v_) generated a new sub-band-gap level in the proximity of the Fermi level of BiVO_4_ which can affect photocatalytic efficiency [33]. It is known that the oxygen vacancy-related V^4+^ is paramagnetic. Thus, the relative density of oxygen vacancies of the BiVO_4_ samples at various calcination temperatures can be quantified by comparing their relative amount of V^4+^ using electron paramagnetic resonance (EPR) spectroscopy which is highly sensitive for paramagnetic states even at extremely low concentrations. Figure 8a compares the EPR spectra of all samples, whereby a signal centered at g = 1.978 corresponds to the g value reported for paramagnetic V^4+^ is observed for each sample [34]. Figure 8b shows the corresponding concentration of paramagnetic V^4+^ centers through double integral of EPR signal for each catalyst. The observed signals were strongly influenced by the calcination temperature. Based on Figure 8a, with an increase in calcination temperature up to 450 °C the EPR signals for paramagnetic V^4+^ center increased which might be due to the phase transformation from the tetragonal to monoclinic phase. However, a further increase of calcination temperature to 600 °C diminishes the paramagnetic V^4+^ center density, as indicated by the weaker EPR signal (Figure 8a) and its defect concentration (Figure 8b) of BV-600 relative to that of BV-450. This might be due to the reorientation of the crystal lattice that starts to occur at temperatures above 450 °C, i.e., BV-525 and BV-600 [32]. The existence of defect sites in the BiVO_4_ photocatalyst has been reported to be crucial in enhancing photocatalytic performance for various applications such as water splitting [16,32], solar to fuel [35], and methylene blue degradation [36].

### 2.6. Photocatalytic Performance

**Figure 9:**
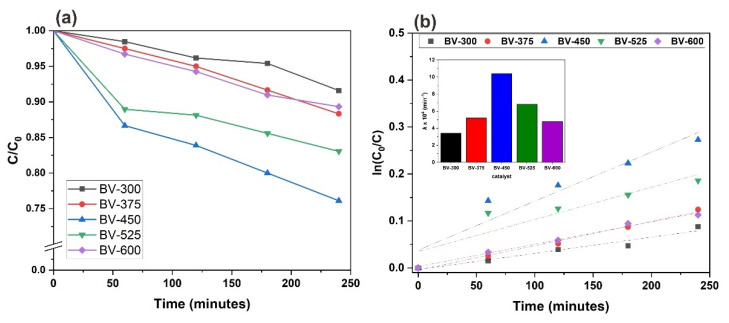
(**a**) Photocatalytic degradation of POME. (**b**) Pseudo-first-order linear plots of ln(C_0_/C) versus irradiation time for the degradation kinetics of POME using BiVO_4_ photocatalyst with varying calcination temperature. Inset: the apparent first-order rate constant (*k*) of BiVO_4_ catalysts with different calcination temperatures.

The photocatalytic activity of the synthesized BiVO_4_ photocatalyst with different calcination temperatures was evaluated by degradation of POME waste under 300 W Xenon lamp irradiation (UV-visible light). Calibration of the standard COD solution was conducted prior to evaluate the performance of photocatalysts (Figure S1). The relationship between the degradation results and the reaction time for the BiVO_4_ photocatalyst with different calcination temperatures is shown in Figure 9a. It can be determined that the self-degradation of POME waste is very small in the absence of photocatalyst, which indicates that the photolysis effect is negligible (Figure S2). The adsorption of waste POME on the BiVO_4_ photocatalyst in the dark (in the absence of a light source) was also evaluated. After 240 min, the COD concentration in POME waste changed very little, indicating that the degradation of POME waste by BiVO_4_ photocatalyst was mainly due to photodegradation rather than adsorption. Figure 9a also shows that the degradation efficiency appears to be following a hyperbolic trend. This may indicate that photocatalyst deactivation has been occurred, most likely through the adsorption of intermediate organic species at the active site, consequently the number of active sites decreases with irradiation time.

A linear plot of ln(C_0_/C) versus irradiation time t (min) for POME waste degradation is shown in Figure 9b where the value of *k* is determined from the linear slope. The apparent reaction rate constant *k* was used to evaluate the degradation rate as shown in Figure 9b (inset). BV-450 exhibited the highest reaction rate constant and it is about three times higher than BV-300. The value of R^2^ obtained from 0.84 to 0.99, indicates very good linearity which also confirms that POME effluent degradation follows first-order reaction kinetics. It should be noted that the increased photocatalytic activity of the BV-450 photocatalyst could be attributed to the smaller grain size, mesoporous structure, and higher specific surface area. Smaller grain size and mesoporous structure can reduce the migration length of photogenerated charge carriers and further reduce the rate of photogenerated electron-hole pair recombination. The higher specific surface area gives more active sites, which is favorable for the catalytic reaction.

The photocatalytic degradation rates and reaction rate constant of BiVO_4_ photocatalysts were found to decrease in the order BV-450 > BV-525 > BV-375 > BV-600 > BV-300. The increase in photocatalytic activity can be ascribed to the efficient separation of the photogenerated charge carriers in the monoclinic phase as a result of the increase in calcination temperature up to 450 °C. Another reason for this result may be due to the fact that photocatalyst BV-450 exhibits the highest V^4+^ center paramagnetic defect sites (Figure S3) which mimicked the photocatalytic performance profile, clearly illustrating the involvement of these defect sites during the photocatalytic process. The vanadium vacancy is mainly located on the surface and traps electrons and facilitates charge separation efficiently and thus boosting photocatalytic performance [37]. On the other hand, the poor photocatalytic behavior of photocatalysts other than BV-450 might be due to particle agglomeration which reduces the surface-to-volume ratio and limits the amount of incoming light radiation [38].

Furthermore, the photocatalytic performance and reaction rate kinetics of BV-450 are still the lowest among other semiconductor-based photocatalysts (Table S1 in Supplementary Materials) but it can be further improved by considering and modifying several physiochemical properties of BiVO_4_ photocatalyst including the density of defect sites, band-gap energy, phase composition, morphology, and crystal facet engineering. Based on the concept of photocatalytic degradation of organic pollutants, the following mechanisms can be proposed for photocatalytic degradation of POME using the BiVO_4_ photocatalyst (Equations (1)–(9)):

BiVO_4_ + *hv* → BiVO_4_ (e^−^_CB_ + h^+^_VB_) (1)

e^−^_CB_ + O_2,ads_ → •O_2_^−^ (2)

•O_2_^−^ + H_2_O → O_2_H• + OH^−^ (3)

2O_2_H• → H_2_O_2_ + O_2_ (4)

H_2_O_2_ + *hv* → 2OH• (5)

OH^−^ + h^+^ → OH• (6)

h^+^ + H_2_O_ads_ → OH• + H^+^ (7)

OH• + Complex organics (POME) → POME degradation(8)

h^+^ + e^−^ → heat (recombination) (9)

**Figure 10:**
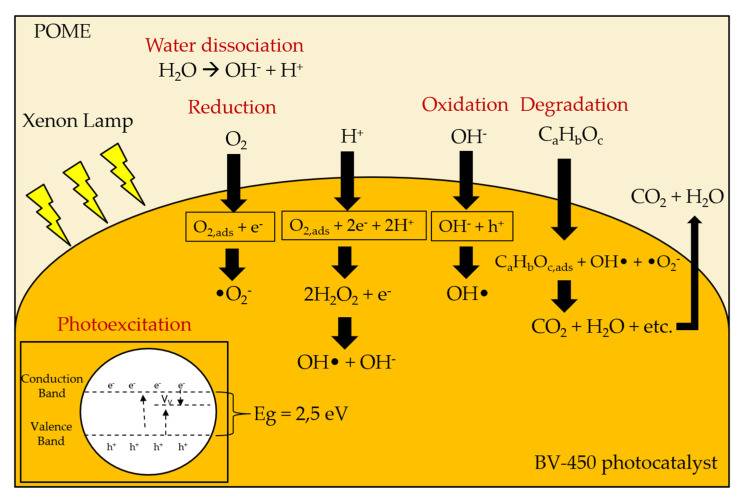
Proposed mechanisms of photocatalytic POME removal using BV-450 photocatalyst. Adapted from Cheng et al. [39].

A possible photocatalytic mechanism for enhancing the photocatalytic performance of the BV-450 photocatalyst can be proposed by taking into account the charge transfer process (Figure 10). Light excitation from the photocatalyst of BiVO_4_ nanoparticles generates energetic electrons (e^−^_CB_) and positively charged holes (h^+^_VB_). The existence of vanadium vacancy defects creates a new sub-band-gap energy level in between the valence band and conduction band. These levels act as electron traps that exhibit charge separation more effectively. Photogenerated holes can react directly with H_2_O and OH^−^ to produce hydroxyl radicals (OH•), which play an important role in the degradation of POME waste. Meanwhile, photogenerated electrons will be captured by O_2_ which is adsorbed on the surface of BiVO_4_ which acts as an active site to produce superoxide radicals •O_2_^−^. Ultimately, the active species (holes, superoxide radicals, and hydroxyl radicals) oxidize the POME molecules adsorbed on the active site of the BV-450 photocatalyst. The hydroxyl radical OH• is a very strong and non-selective oxidant (E° = +2.80 V) which causes partial or complete degradation of some organic chemicals [25]. In addition, the higher density of the V^4+^ center’s defect sites of the BV-450 photocatalyst provide an easier path for charge carrier transport along the granule, leading to an increase in the photogenerated charge carrier separation efficiency. Ultimately, these findings fulfill the gap of BiVO_4_-based photocatalyst applications for photocatalytic degradation of various organic pollutants, in particular, POME waste (Table S2).

## 3. Materials and Methods

### 3.1. Synthesis of Materials

All chemicals used in this work were of analytical grade and used as received without further purification. In a typical preparation process, 0.01 mol of Bi(NO_3_)_3_·5H_2_O and 0.02 mol of citric acid (CA) were dissolved in 40 mL of 2.0 mol/L HNO_3_ and stirred for about 30 min until a clear solution was obtained (denoted as a solution A). A total of 0.01 mol of NH_4_VO_3_ and 0.02 mol of CA were dissolved in 40 mL of 1 mol/L NH_3_ and stirred until a transparent orange solution was obtained (denoted as solution B). After the addition of solution A to solution B, a uniform transparent dark green solution was obtained (denoted as solution C). The obtained sol was stirred for about 30 min until a light blue solution was obtained and then heated up to 75 °C for water evaporation. The formed gel was calcined in air at different temperatures ranging from 300 to 600 °C for 3 h with a heating rate of 2 °C min^−1^. Samples are labeled BV-x, where BV and x are attributed to BiVO_4_ and calcination temperature, respectively.

### 3.2. Characterization

The crystal structures of the catalysts were determined in air by X-ray diffraction (XRD, Bruker D8 Advance, Billerica, MA, USA) using Cu-Ka radiation (λ = 0.15406 nm). The crystallite size was estimated from the Scherrer equation using full width at half the maximum height of the peak at 2θ = 28.9°. Raman spectra were recorded using a Raman spectrometer (Bruker-Senterra, Billerica, MA, USA) with a 514 nm argon-ion laser. The morphology and size of the particles were obtained using a scanning electron microscope (SEM, SU3500, Hitachi High-Technologies Corporation, Tokyo, Japan) and a transmission electron microscope (TEM, HT7700, Hitachi High-Technologies Corporation, Tokyo, Japan) coupled with energy-dispersive X-ray (EDX, SU3500, Hitachi High-Technologies Corporation, Tokyo, Japan). Nitrogen physisorption measurements were carried out at 77 K with a Micromeritics TriStar II 3020 analyzer (Micromeritics Instrument Corporation, Norcross, GA, USA). Prior to the adsorption measurement, the samples were degassed at 150 °C for 3 h. The specific surface areas were calculated according to the Brunauer–Emmett–Teller (BET) method and porosity parameters were determined according to the Barrett–Joyner–Halenda (BJH) method. Diffuse reflectance spectra were recorded using UV-Vis spectrophotometer (Thermo Scientific Evolution 200, Waltham, MA, USA) and converted to absorbance using the Kubelka–Munk method with BasSO_4_ as a reference. The existence of defects was evaluated using electron paramagnetic resonance (EPR) spectroscopy (Bruker ELEXSYS E-500, Billerica, MA, USA). EPR measurements were conducted at 9.419 GHz (X-band). The microwave power, the modulation amplitude, and the temperature were set at 2 mW, 5 G, and 120 K, respectively.

### 3.3. Photocatalytic Activity

Fresh POME was collected from PTPN VII Palm Oil Mill in Bogor, Indonesia. For the study of photocatalytic degradation of POME, fresh POME was filtered to remove solid suspension then diluted with deionized water. Filtration and dilution steps were necessary since the fresh POME in its existing form was impenetrable to light sources.

The BiVO_4_ samples were evaluated via photocatalytic degradation of POME under Xenon light irradiation. The photocatalytic reaction setup is shown in Figure S4. Typically, 0.5 g of a photocatalyst was added into 500 mL of pretreated POME (COD level ranging from 200 to 300 ppm) and stirred in the dark for 30 min to achieve an adsorption–desorption equilibrium. At the same time, a high purity grade of O_2_ gas was bubbled into the POME wastewater at a flow rate of 70 mL min^−1^ to provide an O_2_ source [40]. The suspension was then irradiated using a 300 W Xenon lamp and fitted into a cooling water compartment that was externally circulated with water, in order to remove light source-dissipated heat. Each photocatalytic reaction was conducted for 240 min. At irradiation intervals of every 60 min, 10 mL of the suspension was taken and filtered through a 0.22 µm syringe filter nylon to remove suspended photocatalyst agglomerates. The POME concentration profile was obtained from the COD analysis using Hach DRB-200 COD reactor with the aid of Shimadzu 1800 Spectrophotometer. The reaction rate constant (*k*) was then determined assuming pseudo-first-order kinetics using Equation (10) as follows:

(10) $$ln\left(\frac{C_{0}}{C}\right)=kt$$

where $C_{0}$ is the initial COD concentration of POME waste after being stirred in the dark for 30 min (mg/L), and C is the COD concentration of POME waste at irradiation time *t* (mg/L), *k* is the apparent reaction rate constant (min^−1^).

## 4. Conclusions

In this study, the BiVO_4_ photocatalyst was successfully synthesized using the sol-gel method and it was optimized by different calcination temperatures. The effect of calcination temperature in the range of 300–600 °C on the alteration of structure, phase transformation, density of defect sites, and morphology of the BiVO_4_ photocatalyst was studied. The photocatalytic performance of BiVO_4_ was evaluated by the degradation of POME under UV-visible light irradiation. The highest photocatalytic activity of about 24% removal efficiency with the apparent rate constant of 1.04 × 10^−3^ min^−1^ was obtained for BV-450 mainly due to the high density of vanadium vacancy defect sites. The vanadium vacancy could trap photogenerated electrons and promote charge separation efficiently; hence this significantly boosted the photocatalytic performance. The additional absorption band energy due to the existence of vanadium vacancy defect sites and widening of the optical absorption spectrum of BV-450 in the visible range with a monoclinic scheelite structure paves the way for efficient visible light-driven photocatalytic activity for POME degradation.

## Figures and Tables

**Table 1:** Crystallite size and crystallinity percentage of the BiVO_4_ photocatalyst with different calcination temperatures.

Photocatalyst	Crystallite Size (nm)	Crystallinity (%)
BV-300	23.79	93.9
BV-375	21.58	52.1
BV-450	23.89	94.6
BV-525	25.68	99.4
BV-600	26.06	89.1

**Table 2:** Atomic composition quantification using EDX analysis of the BiVO_4_ photocatalyst with different calcination temperatures.

Photocatalyst	Atomic, %			Bi/V Ratio
	Bi	V	O	
BV-300	22.71	34.70	42.59	0.65
BV-375	21.75	29.62	48.63	0.73
BV-450	15.57	27.71	54.72	0.56
BV-525	21.90	30.39	47.71	0.72
BV-600	22.68	33.73	43.60	0.67

**Table 3:** Specific surface area, pore volume, and pore size of BiVO_4_ photocatalyst with different calcination temperatures.

Photocatalyst	S_BET_ (m^2^ g^−1^)	Pore Volume (cm^3^ g^−1^)	Pore Size (nm)
BV-300	1.800	0.017	20.0
BV-375	4.040	0.027	22.0
BV-450	3.450	0.016	19.8
BV-525	1.500	0.009	19.5
BV-600	1.133	0.007	20.3

**Table 4:** Band-gap energy of the BiVO_4_ photocatalyst with different calcination temperatures.

Photocatalyst	E_g_ (eV)
BV-300	2.47
BV-375	2.48
BV-450	2.50
BV-525	2.46
BV-600	2.44

## Supplementary Materials

The following are available online, Figure S1: Calibration curve of standard COD solution, Figure S2: Photocatalytic degradation of POME and pseudo-first-order linear plots of ln(C_0_/C) versus irradiation time for the degradation kinetics of POME using BV-450 with varying operating conditions consisting the presence of BV-450 catalyst, oxygen purging, and irradiation with Xenon lamp. Inset: the apparent first-order rate constant (*k*) of BV-450 catalyst with different operating conditions, Figure S3: Relationship between the apparent first-order rate constant (*k*) and normalized double integrated intensity of EPR spectra, Figure S4: Schematic representation diagram of the photocatalytic reactor and actual photocatalytic reactor setup, Table S1: Photocatalytic performance for POME degradation using various metal oxide-based semiconductor photocatalysts, Table S2: Photocatalytic performance of neat BiVO_4_ photocatalyst for various pollutant degradation.

## Sample Availability

Samples of the compounds are available from the authors.
