# Supplementary material for: Photocatalytic Degradation of Palm Oil Mill Effluent (POME) Waste Using BiVO4 Based Catalysts
Source: Molecules. 2021 Oct 15;26(20):6225. doi: 10.3390/molecules26206225 (PMC8537951; doi:10.3390/molecules26206225)
Supplement: Supplementary file 1 [file molecules-26-06225-s001.zip › molecules-1418276-supplementary.pdf]

molecules

SUPPLEMENTARY INFORMATION

Photocatalytic Degradation of Palm Oil Mill Effluent (POME) Waste Using $BiVO_4$ Based Catalysts

Wibawa Hendra Saputera[1,2,3,*], Aryan Fathoni Amri[1], Rino R. Mukti [2,4,5], Veinardi Suendo[4,5], Hary Devianto[1,3], Dwiwahju Sasongko[1,3]

1 Research Group on Energy and Chemical Engineering Processing System, Department of Chemical Engineering, Faculty of Industrial Technology, Institut Teknologi Bandung, Jl. Ganesha no. 10, Bandung 40132, Indonesia; aryanfathoni@gmail.com (A.F.A.); hardev@che.itb.ac.id (H.D.) sasongko@che.itb.ac.id (D.S.)
2 Center for Catalysis and Reaction Engineering, Institut Teknologi Bandung, Jl. Ganesha no. 10, Bandung 40132, Indonesia
3 Research Center for New and Renewable Energy, Institut Teknologi Bandung, Jl. Ganesha no. 10, Bandung 40132, Indonesia
4 Division of Inorganic and Physical Chemistry, Faculty of Mathematics and Natural Sciences, Institut Teknologi Bandung, Jl. Ganesha no. 10, Bandung 40132, Indonesia; rino@chem.itb.ac.id (R.R.M.); vsuendo@chem.itb.ac.id (V.S.)
5 Research Center for Nanoscience and Nanotechnology, Institut Teknologi Bandung, Jl. Ganesha no. 10, Bandung 40132, Indonesia
* Correspondence: hendra@che.itb.ac.id (W.H.S); Tel.: +62-821-1768-6235

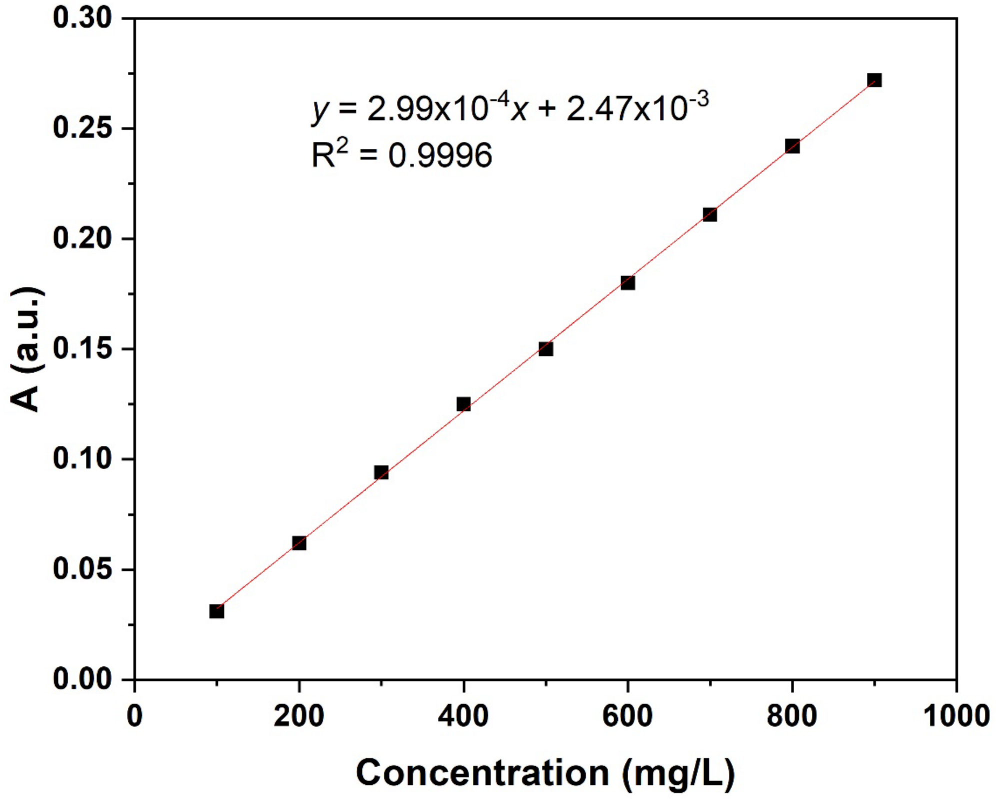

Figure S1. Calibration curve of standard COD solution.

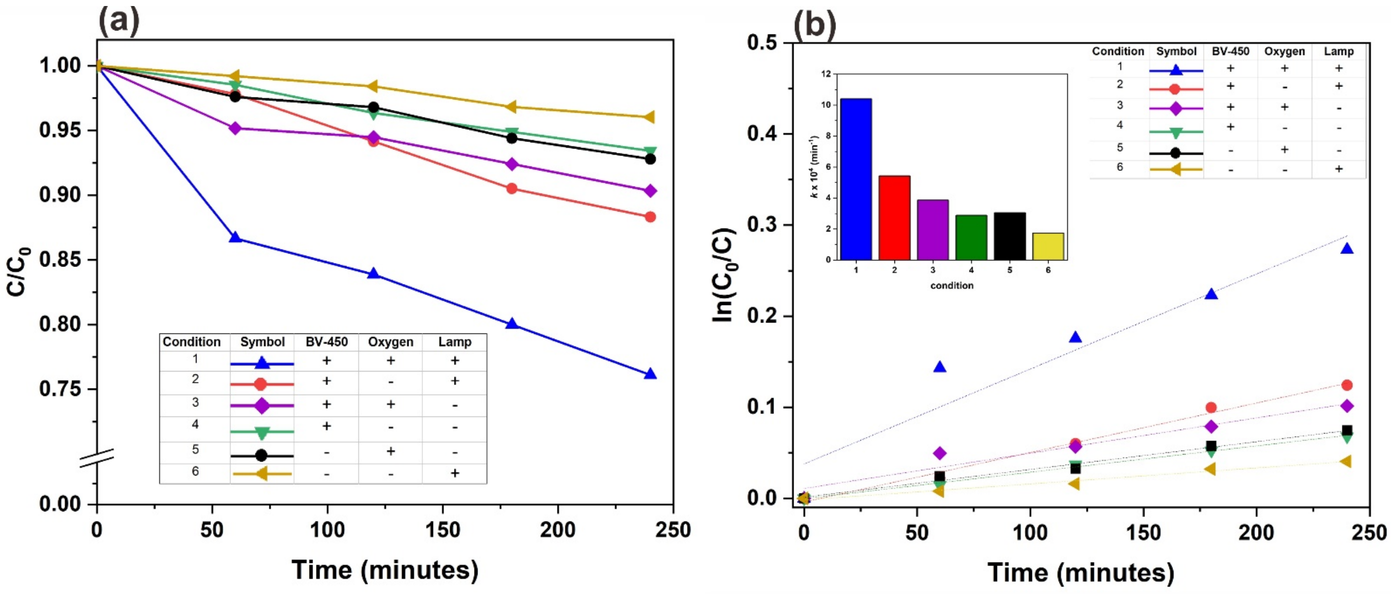

Figure S2. (a) Photocatalytic degradation of POME (b) Pseudo-first-order linear plots of ln(C_0/C) versus irradiation time for the degradation kinetics of POME using BV-450 with varying operating conditions consisting the presence of BV-450 catalyst, oxygen purging, and irradiation with Xenon lamp. Inset of (b): the apparent first-order rate constant (k) of BV-450 catalyst with different operating conditions.

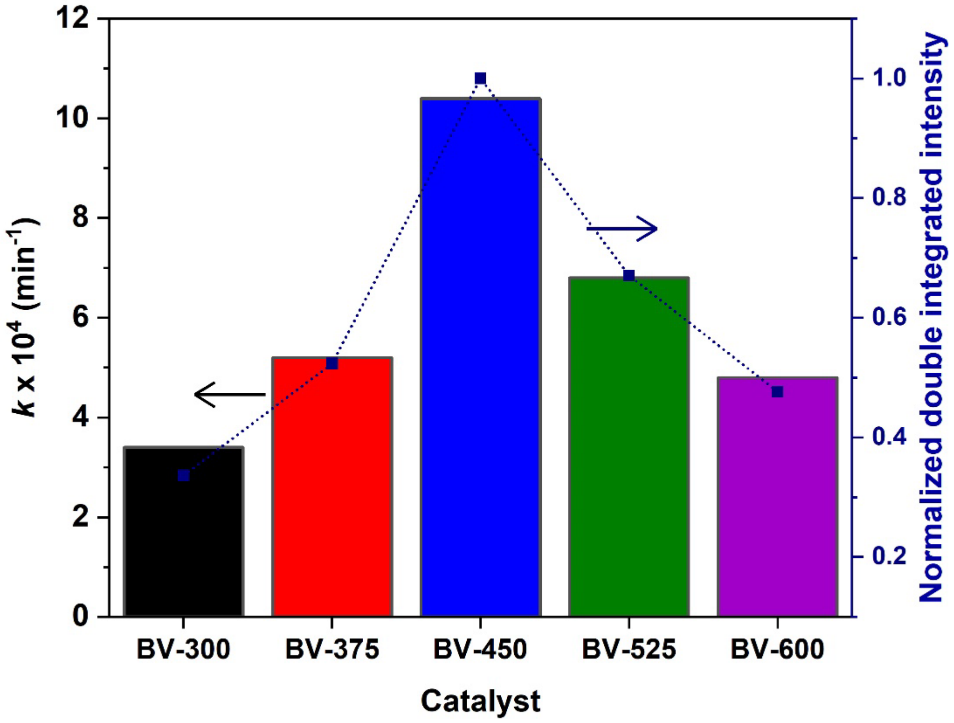

Figure S3. Relationship between apparent first order rate constant (*k*) and normalized double integrated intensity of EPR spectra.

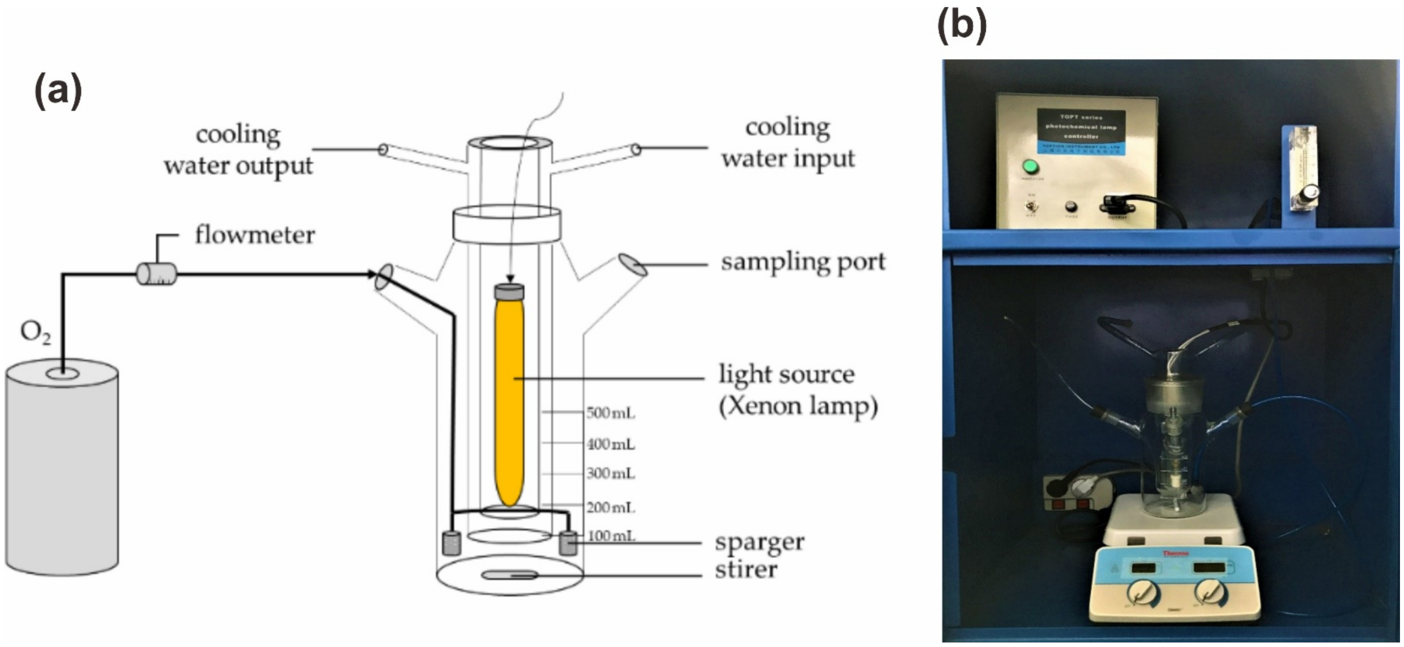

Figure S4. (a) Schematic representation diagram of photocatalytic reactor (b) Actual photocatalytic reactor setup.

Table S1. Photocatalytic performance for POME degradation using various metal oxide-based semiconductor photocatalysts

Photocatalyst	Light Source	Catalyst Loading	Photocatalytic performance	Ref.
TiO_2	UV Fluorescent tube (20 W)	0.01 g/L	COD removal: 97% (42 min)<br>k = n.a.	[1]
TiO_2	UV lamp (100 W)	1.0 g/L	COD removal: 52% (4 h)<br>$k = 2.90 \times 10^{-3}$ min^{-1}	[2]
TiO_2	UV lamp (100 W)	1.04 g/L	COD removal: 55% (4 h)<br>k = n.a.	[3]
TiO_2 anatase	Solar light	0.1 g/L	COD removal: 88% (5 h)<br>k = n.a.	[4]
ZnO	Mercury lamp (100 W)	1.0 g/L	COD removal: 50% (4 h)<br>$k = 3.12 \times 10^{-3}$ min^{-1}	[5]
ZnO-PEG	UV lamp (15 W)	0.5 g/L	COD removal: 94% (n.a.)<br>k = n.a.	[6]
$CaFe_2O_4$	Xenon lamp (500 W)	0.75 g/L	COD removal: 69% (8 h)<br>$k = 2.71 \times 10^{-3}$ min^{-1}	[7]
$BiVO_4$	Xenon lamp (300 W)	1.0 g/L	COD removal: 24% (4 h)<br>$k = 1.04 \times 10^{-3}$ min^{-1}	This work

Table S2. Photocatalytic performance of neat $BiVO_4$ photocatalyst for various pollutants degradation.

Pollutant	Light Source	Photocatalyst properties	Pollutant concentration /Catalyst Loading	Photocatalytic performance	Ref.
Palm oil mill effluent (POME)	Xenon lamp (300 W)<br>λ: 200-800 nm	UV-Vis: $E_g = 2.50$ eV<br>$S_{BET} = 3.45$ m^2/g<br>EPR: $V_{vacancy}$ at g=1.978<br>SEM: small granules<br>XRD: Monoclinic	200 ppm/ 1.0 g/L	COD removal: 24% (4 h)<br>$k = 1.04 \times 10^{-3}$ min^{-1}	This work
Methylene blue (MB)	Tungsten halogen (400 W)<br>$\lambda > 400$ nm	UV-Vis: $E_g = 2.33$ eV<br>$S_{BET} = 8.80$ m^2/g<br>SEM: nanoplate<br>XRD: Monoclinic	10 ppm/ 1.0 g/L	MB removal: 98% (2 h)<br>$k = 3.18 \times 10^{-2}$ min^{-1}	[8]
Methyl orange (MO)	Xenon lamp (300 W)<br>$\lambda > 400$ nm	UV-Vis: $E_g = 2.45$ eV<br>$S_{BET} = 8.40$ m^2/g<br>SEM: porous spherical<br>XRD: Monoclinic	3.27 ppm/ 0.5 g/L	MO removal: 84% (2 h)<br>k = n.a.	[9]
Rhodamine Blue (RhB)	Xenon lamp (500 W)<br>$\lambda > 400$ nm	UV-Vis: $E_g = 2.50$ eV<br>SEM: ellipsoidal<br>XRD: Monoclinic	10 ppm/ 0.4 g/L	RhB removal: 50.5% (2.5 h)<br>k = n.a.	[10]
Carbamazepine (CBZ)	Xenon lamp (300 W)<br>$\lambda > 420$ nm	UV-Vis: $E_g = 2.32$ eV<br>$S_{BET} = 1.04$ m^2/g<br>SEM: hexagonal/cubic<br>XRD: Monoclinic	10 ppm/ 1.0 g/L	CBZ removal: 2% (2 h)<br>$k = 1.20 \times 10^{-4}$ min^{-1}	[11]

References

1. Haji Alhaji, M.; Sanaullah, K.; Fong Lim, S.; Ragai Henry Rigit, A.; Hamza, A.; Khan, A. Modeling and optimization of photocatalytic treatment of pre-treated palm oil mill effluent (POME) in a UV/TiO2 system using response surface methodology (RSM). *Cogent Engineering* **2017**, *4*, 1382980, doi:10.1080/23311916.2017.1382980.
2. Ng, K.H.; Cheng, C.K. A novel photomineralization of POME over UV-responsive TiO2 photocatalyst: kinetics of POME degradation and gaseous product formations. *RSC Advances* **2015**, *5*, 53100-53110, doi:10.1039/C5RA06922J.
3. Ng, K.H.; Cheng, C.K. Photocatalytic degradation of palm oil mill effluent over ultraviolet-responsive titania: Successive assessments of significance factors and process optimization. *Journal of Cleaner Production* **2017**, *142*, 2073-2083, doi:https://doi.org/10.1016/j.jclepro.2016.11.077.
4. Kanakaraju, D., Liyana Binti Ahmad, N., Binti Mohd Sedik, N., Gan Hsien Long, S., Meng Guan, T., & Ying Chin, L. . Performance of Solar Photocatalysis and Photo-Fenton Degradation of Palm Oil Mill Effluent. *Malaysian Journal of Analytical Science* **2017**, *21*, 996-1007.
5. Ng, K.H.; Cheng, C.K. Photo-polishing of POME into CH4-lean biogas over the UV-responsive ZnO photocatalyst. *Chemical Engineering Journal* **2016**, *300*, 127-138, doi:https://doi.org/10.1016/j.cej.2016.04.105.
6. Zainuri, N.Z.; Hairom, N.H.H.; Sidik, D.A.B.; Desa, A.L.; Misdan, N.; Yusof, N.; Mohammad, A.W. Palm oil mill secondary effluent (POMSE) treatment via photocatalysis process in presence of ZnO-PEG nanoparticles. *Journal of Water Process Engineering* **2018**, *26*, 10-16, doi:https://doi.org/10.1016/j.jwpe.2018.08.009.
7. Charles, A.; Khan, M.R.; Ng, K.H.; Wu, T.Y.; Lim, J.W.; Wongsakulphasatch, S.; Witoon, T.; Cheng, C.K. Facile synthesis of CaFe2O4 for visible light driven treatment of polluting palm oil mill effluent: Photokinetic and scavenging study. *Science of The Total Environment* **2019**, *661*, 522-530, doi:https://doi.org/10.1016/j.scitotenv.2019.01.195.
8. Xu, C.; Zhu, G.; Wu, J.; Liang, J. Template-Free Hydrothermal Synthesis Different Morphologies of Visible-Light-Driven BiVO4 Photocatalysts. *Journal of Nanoscience and Nanotechnology* **2014**, *14*, 4475-4480, doi:10.1166/jnn.2014.8039.
9. Jiang, H.; Dai, H.; Meng, X.; Zhang, L.; Deng, J.; Liu, Y.; Au, C.T. Hydrothermal fabrication and visible-light-driven photocatalytic properties of bismuth vanadate with multiple morphologies and/or porous structures for Methyl Orange degradation. *Journal of Environmental Sciences* **2012**, *24*, 449-457, doi:https://doi.org/10.1016/S1001-0742(11)60793-6.
10. Lei, B.-X.; Zeng, L.-L.; Zhang, P.; Sun, Z.-F.; Sun, W.; Zhang, X.-X. Hydrothermal synthesis and photocatalytic properties of visible-light induced BiVO4 with different morphologies. *Advanced Powder Technology* **2014**, *25*, 946-951, doi:https://doi.org/10.1016/j.apt.2014.01.014.
11. Wang, B.; Li, P.; Du, C.; Wang, Y.; Gao, D.; Li, S.; Zhang, L.; Wen, F. Synergetic effect of dual co-catalysts on the activity of BiVO4 for photocatalytic carbamazepine degradation. *RSC Advances* **2019**, *9*, 41977-41983, doi:10.1039/C9RA07152K.
